# The roles of phosphorylation of signaling proteins in the prognosis of acute myeloid leukemia

**DOI:** 10.3389/pore.2024.1611747

**Published:** 2024-07-05

**Authors:** Adrienn Márton, Katalin Beáta Veres, Ferenc Erdődi, Miklós Udvardy, Árpád Illés, László Rejtő

**Affiliations:** ^1^ Division of Hematology, Department of Internal Medicine, Faculty of Medicine, University of Debrecen, Debrecen, Hungary; ^2^ Kálmán Laki Doctoral School, University of Debrecen, Debrecen, Hungary; ^3^ Laboratory of Cellular Therapy, MEDYAG Kft, Debrecen, Hungary; ^4^ Department of Medical Chemistry, Faculty of Medicine, University of Debrecen, Debrecen, Hungary; ^5^ Department of Hematology, Szabolcs-Szatmár-Bereg County Teaching Hospital, Nyíregyháza, Hungary

**Keywords:** acute myeloid leukemia, retinoblastoma, AKT, ERK, protein phosphorylation

## Abstract

Signaling pathways of Retinoblastoma (Rb) protein, Akt-kinase, and Erk-kinase (extracellular signal-regulated kinase) have an important role in the pathogenesis of acute myeloid leukemia. Constitutive activation of these proteins by phosphorylation contributes to cell survival by regulation of cell cycle, proliferation and proapoptotic signaling processes. According to previous data phosphorylated forms of these proteins represent a worse outcome for cancer patients. We investigated the presence of phosphorylated Rb (P-Rb), Akt (P-Akt) and Erk (P-Erk) proteins by Western blot technique using phospho-specific antibodies in bone marrow or peripheral blood samples of 69 AML patients, 36 patients with myelodysplastic syndrome (MDS) and 10 healthy volunteers. Expression level of PTEN (Phosphatase and tensin homolog) and PHLPP (PH domain and leucine-rich repeat Protein Phosphatase) phosphatases, the negative regulators of Akt kinase pathway were also examined. We tested the effect of these proteins on survival and on the correlation with known prognostic features in AML. We found 46.3% of AML patients had detectable P-Rb, 34.7% had P-Akt and 28.9% had P-Erk protein. 66.1% of patients expressing PTEN, 38.9% PHLPP, 37.2% both PTEN and PHLPP and 32.2% neither PTEN nor PHLPP phosphatases. Compared to nucleophosmin mutation (NPMc) negative samples P-Erk was significantly less in nucleophosmin mutated patients, P-Rb was significantly less in patients’ group with more than 30 G/L peripheral leukocyte count by diagnosis. PHLPP was significantly present in FAB type M5. The expression of P-Rb represented significant better overall survival (OS), while P-Akt represented significantly worse event-free survival (EFS) in unfavorable cytogenetics patients. The presence of both PHLPP and PTEN phosphatases contributes to better OS and EFS, although the differences were not statistically significant. We confirmed significant positive correlation between P-Akt and PHLPP. Assessing the phosphorylation of Rb, Akt and Erk may define a subgroup of AML patients who would benefit especially from new targeted treatment options complemented the standard chemotherapy, and it may contribute to monitoring remission, relapse or progression of AML.

## Introduction

Acute myeloid leukemia (AML) is characterized by clonal malignant proliferation of immature myeloid blast cells. AML may develop by dysregulation of signaling pathways that enhance the proliferation and survival of hematopoietic progenitor cells. Among others, these may include altered activation of oncogenic protein kinase pathways such as mitogen-activated protein kinases (Raf/MEK/Erk or p44/p42) [1], Akt kinase (also termed as protein kinase B, PKB) [2] and dysfunction of tumor suppressor proteins such as retinoblastoma protein (Rb) [3]. These changes may lead to uncontrolled cell growth and attenuate the efficacy of chemotherapeutic treatments contributing to poor prognosis of leukemia patients. The reversible phosphorylation of proteins balanced by protein kinases and protein phosphatases plays a crucial role in the regulation of the above processes [4, 5], therefore selective targeting of these interconverting enzymes in the signaling pathways might represent novel therapeutic possibilities in AML treatment.

Flt3 (Fms-like tyrosine kinase 3) is the most frequently mutated genes in AML [6, 7]. In normal cells, FL-mediated triggering of Flt3 affect the activation of downstream phosphatidylinositol-3-kinase (PI3K)/Akt and Ras/Raf/MEK/Erk pathways [8]. Flt3-internal tandem duplication (ITD) and tyrosine kinase domain (TKD) mutations result in the constitutive activation of Flt3 kinase and its downstream proliferative signaling pathways including the Mitogen-activated protein kinases (MAPKs) and Akt pathway in leukemic blast cells [9–11].

Mitogen-activated protein kinases are members of the serine/threonine specific protein kinase family. MAPKs play an important role in cell proliferation, differentiation, motility and cell death [12, 13]. The MAPK signaling pathways can be activated by several extracellular stimuli including mitogens, growth factors and cytokines [14, 15]. After stimulation, a sequential protein kinase cascade is initiated. In general, the ligand such as epidermal growth factor (EGF) or platelet-derived growth factor (PDGF) binds to their receptor tyrosine kinase that leads to GTP loading and Ras-GTPase activation [16], which recruits Raf to the membrane and results in its activation. Then Raf phosphorylates, thereby activates MEK1/2 which activates Erk1/2 or p44/p42 by phosphorylation of residues Thr202/Tyr204 (Erk1) and Thr185/Tyr187 (Erk2) in the activation loop. The active Erk1/2 translocates to the nucleus [17] and activates various downstream targets including several transcription factors such as Elk-1 [18], c-Fos [19], c-Myc [20]. Inactivation of MAPKs occurs through dephosphorylation by a family of dual-specificity (phospho-Ser/Thr and -Tyr) phosphatases known as mitogen-activated protein kinase phosphatases (MKPs) [21]. Previous data suggest that protein phosphatase-2A (PP2A), one major type of the phospho-Ser/Thr specific protein phosphatase may also take part in the dephosphorylation of Erk1/2 [22, 23].

Akt serine/threonine kinase or PKB plays a crucial role in cell survival by inhibiting apoptosis. Akt phosphorylates and inactivates several proapoptotic proteins, a few examples are Bax, Bad [24] caspase-9 [25], FOXO tumor suppressors [26]. Akt diminishes the cellular level of p53 through Mdm2 activation [27], activates NFκB [28] and CREB [29] transcription factors, thereby increases the transcription of several anti-apoptotic proteins. Akt upregulates the anti-apoptotic Bcl-2 expression through cAMP-response element-binding protein [30]. Akt also plays a role in cell cycle regulation due to preventing glycogen synthase kinase-3beta (GSK-3β) mediated degradation of cyclin D1 [31]. Akt is activated by various growth factors and survival signals that induce the production of phosphatidylinositol (3,4,5)-triphosphate (PIP3) generated by PI3 kinase. PIP3 recruits phosphoinositide-dependent protein kinase 1 (PDK1) and Akt to the plasma membrane, where PDK1 phosphorylates Akt in the activation loop of the kinase domain at Thr308 [32]. Full activation of Akt requires phosphorylation on the regulatory tail at Ser473 by phosphoinositide-dependent protein kinase 2 (PDK2) or mTORC2 [33], which regulates the degree of activation. PTEN phosphatase is a major negative regulator of the PIP3K/Akt pathway [34]. PTEN limits the production of PIP3 by its dephosphorylation. Genetic inactivation of PTEN leads to constitutive activation of the PI3K/Akt pathway [35]. The PH domain and leucine-rich repeat Protein Phosphatase (PHLPP) dephosphorylates Akt directly on Ser473 [36]. Phosphorylation of Akt is also negatively regulated by PP2A [22, 23].

Retinoblastoma protein is the product of a tumor suppressor gene that plays a crucial role in the regulation of differentiation, apoptosis and cell cycle by coordinating the checkpoint in G1/S transition. Loss of Rb function results in cell cycle progression and enhanced proliferation, which has been shown in a variety of human malignancies [37]. The activity of Rb protein is regulated by reversible phosphorylation and dephosphorylation through protein kinases and protein phosphatases. Hypophosphorylated Rb binds the transcription factor E2F, thereby suppresses the transcription of genes controlling cell cycle such as DNA polymerase alpha [38] and cyclin [39] genes. Rb can interact with chromatin remodeling enzymes, such as histone deacetylase (HDAC) through LXCXE amino acid binding motif [40]. Some studies demonstrated that interaction of Rb with HDAC is required for Rb to inhibit E2F [41]. During the cell cycle, Rb is phosphorylated in a sequential manner by distinct cyclin/cyclin dependent kinase (CDK) complexes [42], thereby Rb is converted to a functionally inactive, phosphorylated state which leads to the release of sequestered E2F [43] inducing G1/S transition and the synthesis of genes in S phase [44]. Rb is phosphorylated up to 16 Ser/Thr residues including Ser249/Thr252, Thr356, Thr373, Ser608, Ser612, Ser780, Ser807/Ser811, Thr821 and Thr826. Phosphorylation at these sites may induce structural changes in Rb protein, which leads to the E2F dissociation. Among these sites, Thr821 and Thr826 have special importance because phosphorylation of either residue may result in disjunction of pRb from LXCXE motif-containing interacting partners, like HDAC and viral oncoproteins like T-Ag, Elf-1 and E7 [45]. It suggests that LXCXE binding site is regulated by phosphorylation of Thr 821/826, but not obligatory to inhibit E2F binding [45]. Functional inactivation of Rb can occur from increased phosphorylation resulting from overexpression of cyclins, mutation of CDKs or loss of function of CDK inhibitors [46]. The dephosphorylation process of phospho-Ser/Thr residue of Rb is regulated by protein phosphatase-1 (PP1) and PP2A. Previous data suggest that PP1 is the major Rb phosphatase involved in the direct dephosphorylation of Rb, while PP2A may influence Rb dephosphorylation via an indirect way by regulating the PP1 catalytic subunit (PP1c) [23, 47].

Epigenetic modifications, especially DNA methylation and posttranslational histone modification (methylation and acetylation) represent key mechanisms in regulating gene expression and the pathogenesis of MDS and AML [48, 49]. Increasing evidence has demonstrated that critical epigenetic modifiers, such as DNA methyltransferase enzymes (DNMTs) and chromatin-modifying enzymes - (lysine acetyltransferases (KATs) and histone deacetylases (HDACs), lysine methyltransferases (KMTs) and protein arginine methyltransferases (PRMTs) - are directly or indirectly modulated by PI3K/Akt [50, 51], MEK/Erk [52, 53] and Rb [54] signaling in malignant cells. Following with this several genes with role in PIP3/Akt or Raf/MEK/Erk pathways show altered DNA methylation status in AML [55].

In the present study, we investigated the presence of phosphorylated forms of MAPK, Akt kinase and Rb proteins using phospho-specific antibodies in leukemic blast cells separated from AML patients. The phosphorylated forms of the above-mentioned proteins may be accompanied by increased leukemic cell proliferation and drug resistance with poor prognosis of survival in AML patients. PTEN and PHLPP phosphatases, negative regulators of the Akt kinase, were investigated, too.

## Materials and methods

### Reagents and antibodies

The following reagents and antibodies were used with the provider indicated in brackets:

Ficoll-Paque PLUS (GE Healthcare), MACS human CD34 MicroBead kit (Miltenyi Biotec), protease inhibitor mix (Roche); rabbit anti-human Akt, rabbit anti-human phospho-Ser473-Akt, rabbit anti-human phosphatase and tensin homolog (PTEN), rabbit anti-human Erk, rabbit anti-human phospho-Thr202/Tyr404-Erk antibodies (Cell Signaling Technology); rabbit anti-human PH domain and Leucine rich repeat Protein Phosphatases (PHLPP) antibody (Abcam); mouse anti-human Retinoblastoma (Rb), goat anti-human phospho-Thr821/826-Rb, goat anti-human glyceraldehyde-3-phosphate-dehydrogenase (GAPDH) antibodies (Santa Cruz Biotechnology); horseradish peroxidase-conjugated secondary rabbit antibody (in cases of Akt, phospho-Ser473-Akt, PTEN, Erk, phospho-Thr202/Tyr404-Erk and PHLPP primary antibodies), horseradish peroxidase-conjugated secondary mouse antibody (in case of Rb primary antibody) and horseradish peroxidase-conjugated secondary goat antibody (in cases of phospho-Thr821/826-Rb and GAPDH primary antibodies) (Sigma Aldrich), enhanced chemiluminescence (ECL) reagents, BCA protein assay (Thermo Scientific), NPM1 12th exon (NPM1 F02: 5’-GTT-TCT-TTT-TTT-TTT-TTT-TTT-CCA-GGC-TAT-TCA-AG-3’) (NPM1 R02: 5’-CA-(C/T)-GGT-AGG-GAA-AGT-TCT-CAC-TCT-GC-3’) primers (BioScience Kft), Flt3 ITD 1 (Flt3 14F: 5’-GCA-ATT-TAG-GTA-TGA-AAG-CCA-GC-3’) and 2 (Flt3 15R: 5’-CTT-TCA-GCA-TTT-TGA-CGG-CAA-CC-3’), Flt3 TKD (Flt3 20F: 5'-CCG-CCA-GGA-ACG-TGC-TTG-3') and (Flt3 20R: 5'-GCA-GCC-TCA-CAT-TGC-CCC-3')primers (Merc Kft), AmpliTaq Gold buffer 1 and 2, AmpliTaq Gold polymerase enzyme, MgCl_2_ solution (Applied Biosystems), dNTP (Roche), Betaine solution (Sigma Aldrich), QIAmp kit (Qiagen).

### Patient samples

A total of 69 AML patients were examined, including 57 newly diagnosed, 3 relapsed and 9 refractory AML patients (29 males and 40 females). The median age was 61 years, with a range from 19 to 89 years of age. According to World Health Organization (WHO) classification [56] 29 patients with AML with recurrent genetic abnormalities, 25 patients with AML with myelodysplasia-related changes (MRC), 2 patients with AML related to previous chemotherapy because of preceding malignancies, 2 patients with myeloid sarcoma concurrently with AML were diagnosed. Six examined patients were diagnosed with acute leukemia of ambiguous lineage or mixed phenotype acute leukemia (MPAL). The other patients were not otherwise classified. According to 2017 ELN risk stratification by genetics [57] 7 patients with favorable, 20 patients with unfavorable and 33 patients with intermediate abnormalities were examined. The others (9 patients) had no appraisable cytogenetic result. There were patients in all French-American-British (FAB) classification categories except M7. The majority of patients belong to the M2 (15) or M4 (21) FAB group.

Bone marrow samples from 60 patients, peripheral blood samples from 6 patients were collected. From 3 patients both bone marrow and peripheral blood samples were taken simultaneously. In case of 34 patients, consecutive samples were collected after the induction treatment. Samples were obtained between May 2012 and April 2015. The median follow-up of AML survivors is 48 months (31–56 months) and all but 2 surviving patients have more than 3 years of follow-up from the time of diagnosis. Follow up is failed in case of one patient. The characteristics of AML patients with respect to age, ECOG performance status, WHO classification, French-American-British classification and type of sample collected are shown in Table 1.

**TABLE 1:** Characteristics of AML patients with respect to age, ECOG status, WHO classification, French-American-British (FAB) classification, cytogenetics and type of collected sample.

		Number of patients (69)
AML patients	Newly diagnosed	57
	Relapsed	3
	Refractory	9
Age	Under 60 years	33
	Over 60 years	36
ECOG	0–1	38
	2–3	27
	4–5	4
WHO classification	AML with recurrent genetic abnormalities	29
	AML with myelodysplasia-related changes	25
	AML related to previous chemotherapy	2
	AML with myeloid sarcoma	2
	Mixed phenotype acute leukemia	6
	AML not otherwise classified	5
FAB classification	M0	5
	M1	9
	M2	15
	M3	3
	M4	21
	M5	5
	M6	4
	M7	0
	No appraisable result	2
Cytogenetics	Favorable	7
	Intermediate	33
	Unfavorable	20
	No appraisable cytogenetic result	9
Origine of samples	Bone marrow	60
	Peripheral blood	6
	both	3

In addition, 10 healthy volunteers and 36 myelodysplastic syndrome (MDS) patients were investigated: 19 males and 17 females, the median age was 73 years, with a range from 23 to 86 years of age. According to Revised International Prognostic Scoring System (R-IPSS) score [58] 8 patients with very low, 12 patients with low, 7 patients with intermedier, another 7 patients with high, 2 patients with very high risk categories were diagnosed. Samples were collected with informed consent from patients during the regularly scheduled diagnostic protocol. The procedure of this study meets the directive of The Declaration of Helsinki [59] and is based on the approval of the Research Ethics Committee ETT-TUKEB (file number: RKEB.5824).

Young patients with good clinical performance received “3 + 7” induction therapy. It consisted of ara-C combined with anthracycline daunorubicin or idarubicin according to standard protocol [60]. We administred 45, 60 or 90 mg/m2 daunorubicin [61] depending on the patient’s age and performance according to literature recommendations [62, 63]. Two patients who were not suitable for intensive chemotherapy got hypomethylating agents with nucleoside analog as initial treatment as study participants. High-dose ara-C was administered as consolidation therapy. Acute promyelocytic leukemia (APL) patients received their chemotherapy according to AIDA protocol [64]. Flag-Ida [65] (fludarabine + high-dose ara-C with idarubicin) induction therapy was given the good clinical performance mixed phenotype acute leukemia patients. Flag-Ida, Flag (fludarabine + high-dose ara-C with or without idarubicin), HAM [66] (high-dose ara-C + mitoxantrone) MEC [67] (mitoxantrone + etoposide + ara-C), FLAMSA [68] (fludarabine + amsacrine + ara-C), CLAG-M [69] (cladribine, ara-C, filgastrim, mitoxantrone) or clofarabine was used as salvage protocol. 16 patients underwent an allogeneic transplant. Patients with poor clinical performance got reduced dose ara-C combined with reduced dose daunorubicin or ara-C alone or Hydroxyurea monotherapy. 4 patients got only supportive treatment. Applied treatment regimens are shown in Supplementary Table S1.

### Isolation of primary blasts

Mononuclear cells enriched in leukemic blasts were separated by Ficoll-Paque PLUS density-gradient centrifugation. The samples were heparin-treated and diluted with physiological saline. First 2–3 mL Ficoll-Paque PLUS was added to the centrifuge tube, then 4–6 mL diluted sample was layered carefully on it. This sample was centrifuged at 400 *g* for 20 min at 20 °C. The mononuclear layer was drawn and cells were washed twice with physiological saline by centrifugation at 100 *g* for 5 min at 20 °C. In some cases, CD34^+^ cells were selected by using MACS human CD34 MicroBead kit. After counting, cells were centrifuged at 300 *g* for 10 min. Then cells were suspended in 300 μL phosphate-buffered saline (PBS) and 100 μL of CD34 MicroBeads were added. The cell suspension was incubated for 30 min in the refrigerator (2°C–8 °C). Then cells were washed with 5–10 mL PBS and centrifuged at 300 *g* for 10 min. After aspirating supernatant, cells were resuspended in 500 μL of PBS. Magnetic separation was carried out using MS MACS column placed in the magnetic field of the MiniMACS Separator. The column was rinsed with 500 μL PBS. Then the cell suspension was applied onto the column and flow-through containing unlabeled cells were collected. The column was washed 3 times with 500 μL PBS buffer. Then the column was removed from the separator and placed on a collection tube. After 1 mL of PBS was added onto the column, magnetically labelled cells were flushed out by flatly pushing the plunger into the column.

### Western blotting

Separated mononuclear cells were counted in the Bürker chamber and 5 × 10^6^ cells were collected by centrifugation at 800 *g*. Then cells were washed with PBS and lysed in 150 µL RIPA lysis buffer (50 mM Tris-HCl, 10 g/L Nonident P-40, 10 g/L Na-deoxycholate, 1 g/L Na-dodecyl sulfate (SDS), 0.15 M NaCl, 2 mM EDTA), containing 1 mM DTT, 0.5% protease inhibitor mix and 0.1 μM microcystin-LR. Cell lysis was accelerated by ultrasonic treatment; cell debris was removed by centrifugation (4°C, 16,000 x *g*, 10 min). Lysates were boiled at 100 °C for 10 min with 5x SDS sample buffer (320 mM Tris-Cl pH 6.8, 10% SDS, 50% glycerol, 25% β-mercaptoethanol, 0.01% bromophenol blue). The protein concentration of the lysates was measured by BCA protein assay at 540 nm in an ELISA Reader (Labsystem Multiscan MS). Equal amounts of cell lysates (100 μg) were loaded onto 10% SDS-polyacrylamide gel and subjected to SDS-PAGE, then transferred to nitrocellulose membrane. Nonspecific binding sites were blocked by room temperature (24 °C) for 1 hour with 5% nonfat dried milk in Tris-Buffered Saline (TBS) containing 0.5% Tween-20 (TBST). Membranes were incubated by 4 °C overnight with primary antibodies (1:1000). The membranes were washed three times with TBST for 10 min, then were incubated by room temperature (24 °C) for 1 hour with horseradish peroxidase-conjugated secondary antibody (1:9000). Then membranes were washed two times with TBST and once with TBS for 10 min. The immunoreactive bands were detected by ECL reagents and imaged with FluorChem FC2 Imager (Alpha Innotech). GAPDH identified with anti-GAPDH (1:9000) was used as a loading control after membrane stripping procedures. Due to the limited amount of samples, each experiment could be repeated two times.

### Membrane stripping

Membranes were washed three times with TBST for 5 min. Then membranes were incubated with stripping buffer (0.5 M Tris-HCl pH 6.8, 10% SDS, 8% β-mercaptoethanol) at 50 °C and shaking at 200 rpm for 30 min. Membranes were washed three times with TBST for 5 min, then membranes were ready for the next cycle of antigen identification.

### Densitometry

For densitometry analysis of the Western blot images, ImageJ 1.47v software was used. The densitometric results of Western blots of p-Rb, p-Akt, p-Erk, PHLPP and PTEN proteins were normalized to the internal control GAPDH. The averages of the expression levels were calculated based on the densitometric analysis of two Western blots for each patient. Relative expression levels of each investigated proteins were considered as relatively high or low depending on their relationship to the median expression level of the full cohort of positive samples for the given protein in order to determine subgroups of patients, in which the relative expression levels are high or low. In the case of PTEN protein a third subgroup of patients considered too, in which the relative expression level of PTEN was lower than the median relative expression level of PTEN in healthy individuals. We also paid special attention if the expression level of protein normalized to GAPDH was above 1 (highest relaively expression level).

### PCR

PCR was used to investigate possible mutations in NPM1 genes, FLT-3 ITD and TKD mutation. The examination was done in central laboratory as part of routine investigation protocol. The results are available in the institutional computer system. The Total DNA for PCR was isolated by QIAmp kit reagent according to the manufacturer’s protocol. The investigated region of the NPM1, and FLT-3 genes was amplified by PCR using specific primers. In case of TKD mutation the PCR product was digested by *Eco* RV enzyme. Nuclease free water was used as a template negative control, DNA isolated from healthy volunteer was used as a negative control. DNA isolated from cancerous or leukemic patients in our institute verified positive for NPM1, and FLT-3 mutation was used as a positive control. Plasmid DNA was used as positive control of digestion. Veriti (Applied Biosystems) PCR machine was used. PCR products were loaded onto 3% agarose gel and subjected to electrophoresis.

### Statistical analysis

Statistical analysis was carried out using SPSS Statistics 26 and Graphpad Prism 9.0.2 software. The distribution of prognostic features between the protein groups were compared by Pearson Chi-Square test or Fisher’s Exact Test. Independent Samples *t*-test were used by comparisation the median expression level of each proteins between different groups of patients. Overall (OS) and event-free survival (EFS) was examined, and survival distributions were estimated by Kaplan-Meier’s method, and comparisons were based on Log Rank (Mantel-Cox) tests. OS was measured in all patients from the time of diagnosis until death. EFS was estimated from the date of diagnosis until failure to achieve CR or disease progression after CR or death from any cause [70]. Spearman correlation tests were used by the correlation examination. Cox proportional hazard model was used to test the impact of the different proteins on survival simultaneously with other prognostic factors. Rb category (phosphorylated or unphosphorylated), and other prognostic features: ECOG performance status (0–1 or 4–5), age at diagnosis over 60 years (yes or no), AML with MRC (yes or no) and underwent an allogenic bone marrow transplantation (ABMT) (yes or no) categories were added to the first model (Supplementary Table S2). PTEN, PHLPP and both phosphatase (yes or no), ECOG performance status (0–1 or 4–5), age at diagnosis over 60 years (yes or no), AML with MRC (yes or no), unfavorable cytogenetics (yes or no) and underwent an ABMT (yes or no) categories were added to the another model (Supplementary Table S3.).

## Results

### Phosphoprotein levels and phosphatases in AML patients

**FIGURE 1:**
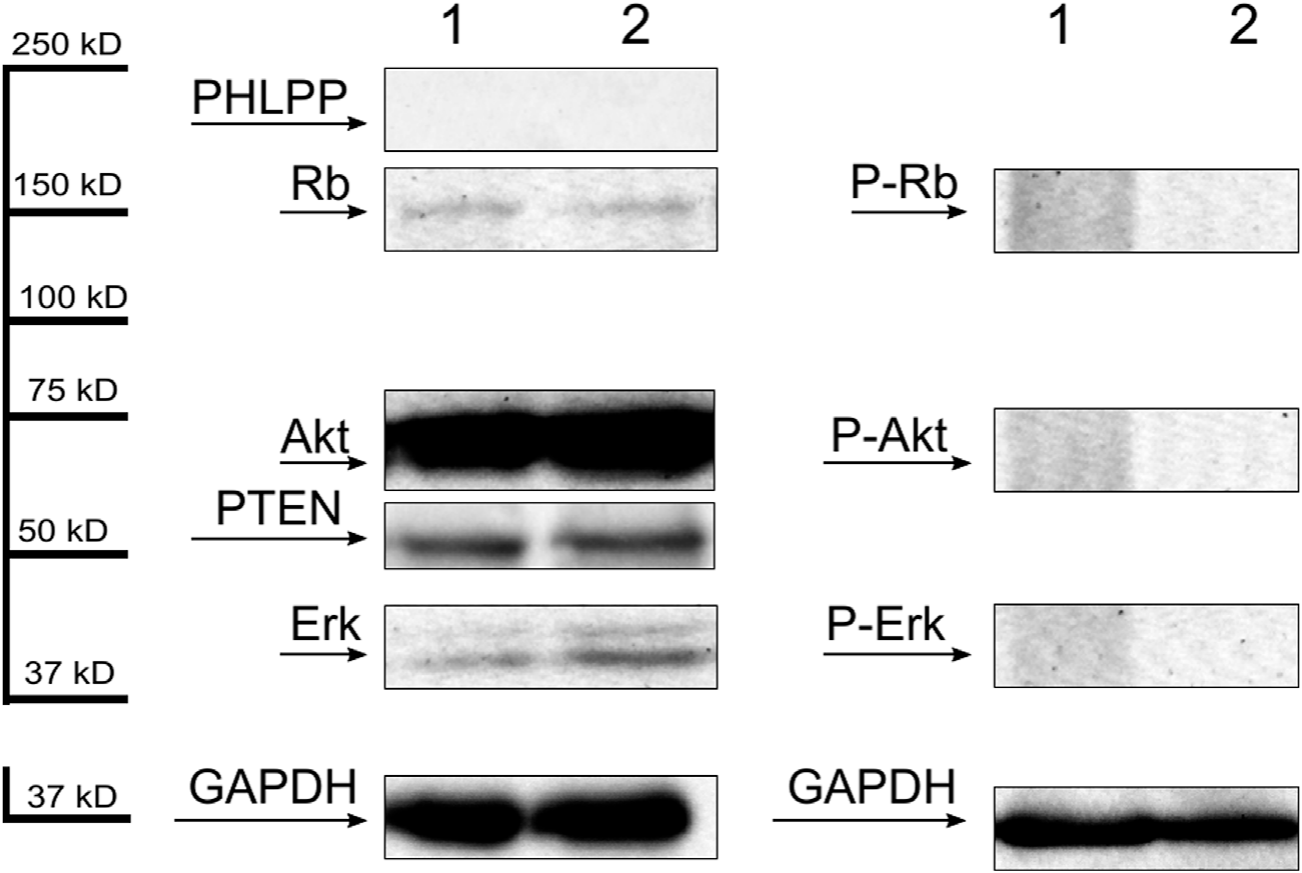
Representative Western blots of dephosphorylated (first panel) and phosphorylated (second panel) forms of Rb, Akt and Erk proteins and PHLPP and PTEN phosphatases (first panel) of mononuclear cells separated from 2 healthy volunteers. GAPDH was used as an internal control.

**FIGURE 2:**
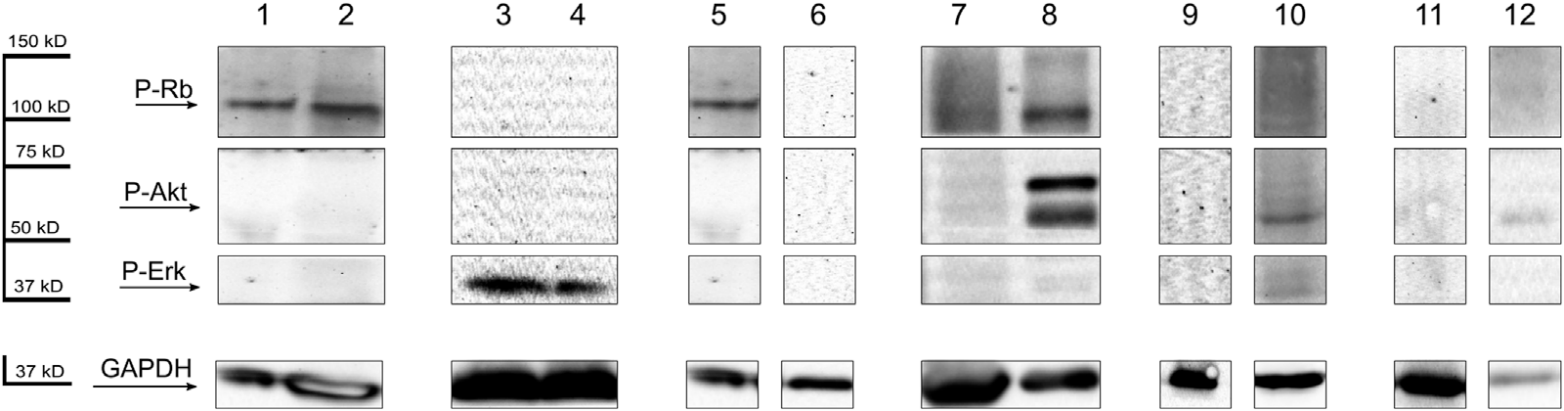
**Lane 1**, magnetic separated CD34^+^ blast cells, and whole mononuclear cells **(lane 2)** after density-gradient separation from bone marrow of the same AML patient. GAPDH was used as an internal control. **Lane 3**, representative Western blots of phospho-Erk of mononuclear cells separated from peripheral blood, and from bone marrow **(lane 4)** of the same AML patient. GAPDH was used as an internal control. **Lane 5**, representative Western blots of P-Rb of mononuclear cells separated from an AML patient at the time of diagnosis, and after chemotherapy treatment **(lane 6)**. GAPDH was used as an internal control. **Lane 7**, Western blots of P-Rb, P-Akt and P-Erk in mononuclear cells separated from an AML patient at the time of diagnosis, and by progression (**lane 8**). GAPDH was used as an internal control. **Lane 9**, Western blots of P-Rb, P-Akt and P-Erk of mononuclear cells separated from a bone marrow of an MDS patient, samples without the presence of AML in the bone marrow. **Lane 10**, samples after the patients progress to AML. GAPDH was used as an internal control. **Lane 11,** Western blots of P-Rb, P-Akt and P-Erk of mononuclear cells separated from a bone marrow of a PNH patient, samples without the presence of AML in the bone marrow. **Lane 12**, samples after the patients progress to AML. GAPDH was used as an internal control.

Thirty-two AML patients (46.3%) had detectable phosphorylated Rb protein level. Akt protein phosphorylation was observed in 24 patients (34.7%). Phospho-Erk protein was detectable in 20 cases (28.9%). Phosphorylation was not detected in mononuclear cells of the 10 healthy individuals, but each showed nonphosphorylated bands of the examined proteins (Figure 1.). Consequently, the observed phosphorylated protein signals must have been attributed to the leukemic cells. The presence of PTEN and PHLPP phosphatases were examined in 59 patients. There were 22 patients (37.2%) expressing both PTEN and PHLPP phosphatases and 19 patients (32.2%) with neither PTEN nor PHLPP expression. Overall, there were 39 patients with detectable PTEN (66.1%) and 23 patients (38.9%) with PHLPP protein. In healthy volunteers, PTEN was detectable in all cases, but PHLPP was not present (Figure 1.). There were no differences in Western blotting results between the magnetic separated CD34^+^ cells and whole mononuclear cells (Figure 2 lane 1 and 2). Therefore, we found the density-gradient separation sufficient for isolating the samples for the assays. There were no differences between results if the samples were collected from blood or bone marrow or obtained simultaneously from 3 patients (Figure 2 lane 3 and 4). Therefore, blood samples may be used in that case if leukemic blasts are detectable in peripheral blood. The presented patient has about 90% blast infiltration in bone marrow and 67% blast cells in peripheral blood.

### Distribution of phosphoproteins and phosphatases among patients grouped by World Health Organization (WHO) classification groups, FAB type, cytogenetic, FLT3-and nucleophosmin mutation status, leukocyte count and percentage of bone marrow blasts at diagnosis

The distribution of phosphoproteins and phosphatases among patients grouped by World Health Organization (WHO) classification, French-American-British (FAB) classification, cytogenetics, Fms Related Receptor Tyrosine Kinase 3 (FLT-3) internal tandem duplication (ITD) or tyrosine kinase domain (TKD) and nucleophosmin (NPMc) mutation status, leukocyte count and percentage of bone marrow blasts at diagnosis is shown in Tables 2, 3.

**TABLE 2:** Comparison of clinical features–World Health Organization (WHO) classification, French-American-British (FAB) classification, cytogenetics, Fms Related Receptor Tyrosine Kinase 3 (FLT-3) and nucleophosmin (NPMc) mutation status, leukocyte count and percentage of bone marrow blasts at diagnosis - known as prognostic in AML for each phosphorylated protein group.

			Phosphorylated proteins			
Characteristic		Total number of patients	P-Rb^(Thr826)^	P-Akt^(Ser473)^	P-Erk^(Thr202/Tyr404)^	*p*-value
			32/69	24/69	20/69	
WHO classification	AML with recurrent genetic abnormalities	29	15/29 (51.7)	8/29 (27.5)	5/29 (17.2)	P = NS
	AML with myelodysplasia-related changes	25	13/25 (52)	11/25 (44)	7/25 (28)	P = NS
	AML related to previous chemotherapy	2	0	1/2 (50)	2/2 (100)	P = NS
	Myeloid sarcoma	2	1/2 (50)	1/2 (50)	1/2 (50)	P = NS
	MPAL	6	3/6 (50)	3/6 (50)	2/6 (33)	P = NS
FAB classification	M0	5	2/5 (40)	2/5 (40)	1/5 (20)	P = NS
	M1	9	2/9 (22.2)	2/9 (22.2)	4/9 (44.4)	P = NS
	M2	15	8/15 (53.3)	5/15 (33.3)	2/15 (13.3)	P = NS
	M3	3	1/3 (33.3)	2/3 (66.6)	0	P = NS
	M4	21	11/21 (52.3)	5/21 (23.8)	8/21 (38)	P = NS
	M5	5	3/5 (60)	1/5 (20)	0	P = NS
	M6	4	2/4 (50)	2/4 (50)	1/4 (25)	P = NS
	M7	0	0	0	0	P = NS
Cytogenetics	favorable cytogenetics	7	1/7 (14.2)	1/7 (14.2)	2/7 (28.5)	P = NS
	Intermediate cytogenetics	33	16/33 (48.4)	11/33 (33.3)	7/33 (21.2)	P = NS
	unfavorable cytogenetics	20	10/20 (50)	9/20 (45)	9/20 (45)	P = NS
FLT-3	wild type	33	18/33 (54.5)	13/33 (39.3)	11/33 (33.3)	P = NS
	ITD-heterozygous	5	3/5 (60)	2/5 (40)	1/5 (20)	P = NS
	TKD- heterozygous	6	1/6 (16.6)	2/6 (33.3)	3/6 (50)	P = NS
	ITD/TKD	1	0	0	0	P = NS
NPMc	mutated	17	11/17 (64.7)	5/17 (29.4)	1/17 (5.8)*	*p = 0.009
	not mutated	31	13/31 (41.9)	12/31 (38.7)	13/31 (41.9)*	*p = 0.009
% blast cells in bone marrow at diagnosis	>30%	56	26/56 (46)	18/56 (32)	17/56 (30)	P = NS
leukocyte count at diagnosis	>30 G/L	27	8/27 (29.6)*	7/27 (25.9)	6/27 (22.2)	*p = 0.029

The cells show the number of cases and percentage in parentheses. Pearson Chi-Square test or in case of sample size under 5 Fisher’s Exact Test was used by analysis of distribution of each prognostic features between each phosphorylated protein group.

**TABLE 3:** Comparison of clinical features–World Health Organization (WHO) classification, French-American-British (FAB) classification, cytogenetics, Fms Related Receptor Tyrosine Kinase 3 (FLT-3) internal tandem duplication (ITD) or tyrosine kinase domain (TKD) and nucleophosmin (NPMc) mutation status, leukocyte count and percentage of bone marrow blasts at diagnosis - known as prognostic in AML for each phosphatase group.

			Phosphatases				
Characteristic		Total number of patients	PTEN	PHLPP	Both PTEN and PHLPP	None of phosphatase	*p*-Value
			39/59	23/59	22/59	19/59	
WHO classification	AML with recurrent genetic abnormalities	27	20/27 (74)	14/27 (51.8)	13/27 (48.1)	6/27 (22.2)	P = NS
	AML with myelodysplasia-related changes	19	11/19 (57.8)	4/19 (21)	4/19 (21)	8/19 (42.1)	P = NS
	AML related to previous chemotherapy	2	2/2 (100)	1/2 (50)	1/2 (50)	0	P = NS
	Myeloid sarcoma	2	2/2 (100)	1/2 (50)	1/2 (50)	0	P = NS
	MPAL	5	3/5 (60)	3/5 (60)	3/5 (60)	2/5 (40)	P = NS
FAB classification	M0	5	4/5 (80)	1/5 (20)	1/5 (20)	1/5 (20)	P = NS
	M1	9	7/9 (77.7)	2/9 (22.2)	2/9 (22.2)	2/9 (22.2)	P = NS
	M2	12	7/12 (58.3)	4/12 (33.3)	4/12 (33.3)	5/12 (41.6)	P = NS
	M3	3	2/3 (66.6)	2/3 (66.6)	2/3 (66.6)	1/3 (33.3)	P = NS
	M4	18	11/18 (61.1)	8/18 (44.4)	7/18 (38.8)	6/18 (33.3)	P = NS
	M5	3	3/3 (100)	3/3 (100)*	3/3 (100) **	0	*p = 0.05 **p = 0.043
	M6	4	2/4 (50)	0	0	2/4 (50)	P = NS
	M7	0	0	0	0	0	P = NS
Cytogenetics	favorable cytogenetics	5	4/5 (80)	2/5 (40)	2/5 (40)	1/5 (20)	P = NS
	Intermediate cytogenetics	30	21/30 (70)	15/30 (50)	14/30 (46.6)	8/30 (26.6)	P = NS
	unfavorable cytogenetics	17	9/17 (52.9)	5/17 (29.4)	5/17 (29.4)	8/17 (47)	P = NS
FLT-3	wild type	30	20/30 (66.7)	11/30 (36.6)	11/30 (36.6)	10/30 (33.3)	P = NS
	ITD-heterozygous	5	3/5 (60)	4/5 (80)	3/5 (60)	1/5 (20)	P = NS
	TKD- heterozygous	6	4/6 (66.6)	2/6 (33.3)	2/6 (33.3)	2/6 (33.3)	P = NS
	ITD/TKD	1	0	0	0	1/1 (100)	P = NS
NPMc	mutated	17	12/17 (70.5)	9/17 (52.9)	8/17 (47)	4/17 (23.5)	P = NS
	not mutated	27	17/27 (62.9)	10/27 (37)	10/27 (37)	10/27 (37)	P = NS
% blast cells in bone marrow at diagnosis	>30%	50	32/50 (64)	20/50 (40)	20/50 (40)	17/50 (34)	P = NS
leukocyte count at diagnosis	>30 G/L	25	17/25 (68)	11/25 (44)	10/25 (40)	7/25 (28)	P = NS

The cells show the number of cases and percentage in parentheses. Pearson Chi-Square test or in case of sample size under 5 Fisher’s Exact Test was used by analysis of distribution of each prognostic features between each phosphatase group.

According to WHO classification we found no significant correlation between groups and the presence of phosphorylated protein or phosphatases.

We found no significant correlation between FAB subtypes and the presence of phosphorylated protein or PTEN phosphatase alone. In FAB5 patients both PHLPP and PTEN, and PHLPP alone was significantly more often present (Table 3).

There was no significant correlation between cytogenetics and presence of phosphorylated proteins or phosphatases.

FLT3 mutation status was identified in case of 45 patients. Internal tandem duplication (ITD) was seen in 5 patients, tyrosine kinase domain mutations (TKD) were present in 6 patients. One patient was found to have both ITD and TKD mutation. These differences were not statistically significant, as the number of cases were not sufficient.

Nucleophosmin mutations were identified in 17 patients, while in 31 patients nucleophosmin were not mutated, but 21 patients had no accessible result. Nucleophosmin mutations were significantly less frequently seen in patients with phosphorylated Erk protein (Table 2).

The percentage of bone marrow blasts (>30%) did not correlate with the presence of phosphorylated proteins or phosphatases. The white blood cell (WBC) counts over 30 G/L at diagnosis were seen significantly less frequently in patients with phosphorylated Rb protein (Table 2).

The occurrence of phosphoproteins or phosphatases showed no correlation with the patients’ age, sex and ECOG performance status.

In summary, the distribution of clinical features was generally similar among the groups. There were no significant differences in age, sex, performance status, presence or absence of preceding MDS, cytogenetics, FLT-3 mutation status or FAB type expect for the presence of both PHLPP and PTEN, and PHLPP in the FAB5 group. Regarding leukocyte count at diagnosis in phosphorylated Rb group and nucleophosmin mutation status in phosphorylated Erk group were significant differences found, but among the other patients’ group the distribution of these prognostic features were similar. The distribution of phosphoproteins and phosphatases between the distinct categories were demonstrated in Tables 2, 3.

### Comparison of the expression levels between different groups of patients

In the subgroup of patients with recurrent genetic abnormalities according to WHO classification, most patients have relative expression level of PTEN higher than the median relative expression level of PTEN in health individuals (N = 27/16 Pearson Chi-Square test *p* = 0.029). Phosphorylated Akt had a significantly higher median expression level in AML with recurrent genetic abnormalities compared to the others (N = 29, Independent Samples *t*-test *p* = 0.044), in concordance with this, only one patient had low phospho-Akt level in this subgroup (N = 29/1 Pearson Chi-Square test *p* = 0.026).

In mixed phenotype acute leukemia patients a significantly higher median expression level of phospho-Rb protein was found (N = 6, Independent Samples *t*-test *p* = 0.044).

Among the different FAB subtypes of AML, heterogeneous expression levels have been detected. In FAB0 subgroup, most patients have relative expression level of PTEN lower than the median relative expression level of PTEN in healthy individuals (N = 5/3, Pearson Chi-Square test *p* = 0.042). In FAB1 subgroup, relatively low phospho Akt level was present and no patients have relatively high expression level (N = 9/0, Pearson Chi-Square test *p* = 0.018). In contrast, in FAB2 relatively high expression level was found and no patients had relatively low phospho-Akt level (N = 15/0, Pearson Chi-Square test *p* = 0.039). In FAB4 relatively high PTEN level was detectable in the majority of PTEN-positive patients (N = 11/9, Pearson Chi-Square test *p* = 0.048) In FAB5 relatively high PHLPP expression was found in every PHLPP positive patients (N = 3/3 Pearson Chi-Square test *p* = 0.006) Among FAB6 patients relatively low phospho-Retinoblastoma level was found and only one patient had relatively high phospho-Retinoblastoma level (N = 4/1 Pearson Chi-Square test *p* = 0.026) There was no significant correlation between the expression level of the other proteins and FAB groups.

There was no significant correlation between cytogenetics, FLT3 and Nucleophosmin mutation status, and the expression level of proteins.

A significantly higher median expression level of phospho-Rb protein was found in the patient’s subgroup in which the percentage of bone marrow blasts was above 30% at diagnosis (N = 56, Independent Samples *t*-test *p* = 0.033). In concordance with this, only minority of patients had low phospho-Retinoblastoma level (N = 56/8 Pearson Chi-Square test *p* = 0.031).

In the subgroup of patients with white blood cell (WBC) counts over 30 G/L at diagnosis relatively low phospho-Erk expression level was detected in the majority of patients. Only two patients presented relatively high phospho-Erk expression level (N = 27/2 Pearson Chi-Square test *p* = 0.047). The white blood cell (WBC) counts over 100 G/L at diagnosis show a significant correlation with lower median phospho-Akt expression level (N = 11 Independent Samples *t*-test *p* = 0.033), in accordance with this, no relatively high phospho-Akt level was detected in this subgroup (N = 11/0 Pearson Chi-Square test *p* = 0.019).

### Relationship between PTEN, PHLPP and AKT

We hypothesized that loss of PTEN or PHLPP, or their decreased expression would lead to high Akt phosphorylation in phospho-Akt positive cases. However, the Spearman correlation test showed significant positive correlation between the presence of phosphorylated Akt and PHLPP (r = 0.318, *p* = 0.014). These data suggest that Akt phosphorylation results in consequent elevation of PHLPP expression in leukemic patients, while PHLPP is not detected in healthy patients. We did not find significant correlation between the presence of PTEN and P-Akt, but if the relative expression level of the proteins considered too, highest PTEN level and highest phosphorylated Akt level showed a significant positive correlation (r = 0.26, *p* = 0.047). The exact molecular mechanisms which lead to constitutive Akt phosphorylation in AML are subject of further investigation.

### Relationship of phosphoprotein level to clinical outcome

**FIGURE 3:**
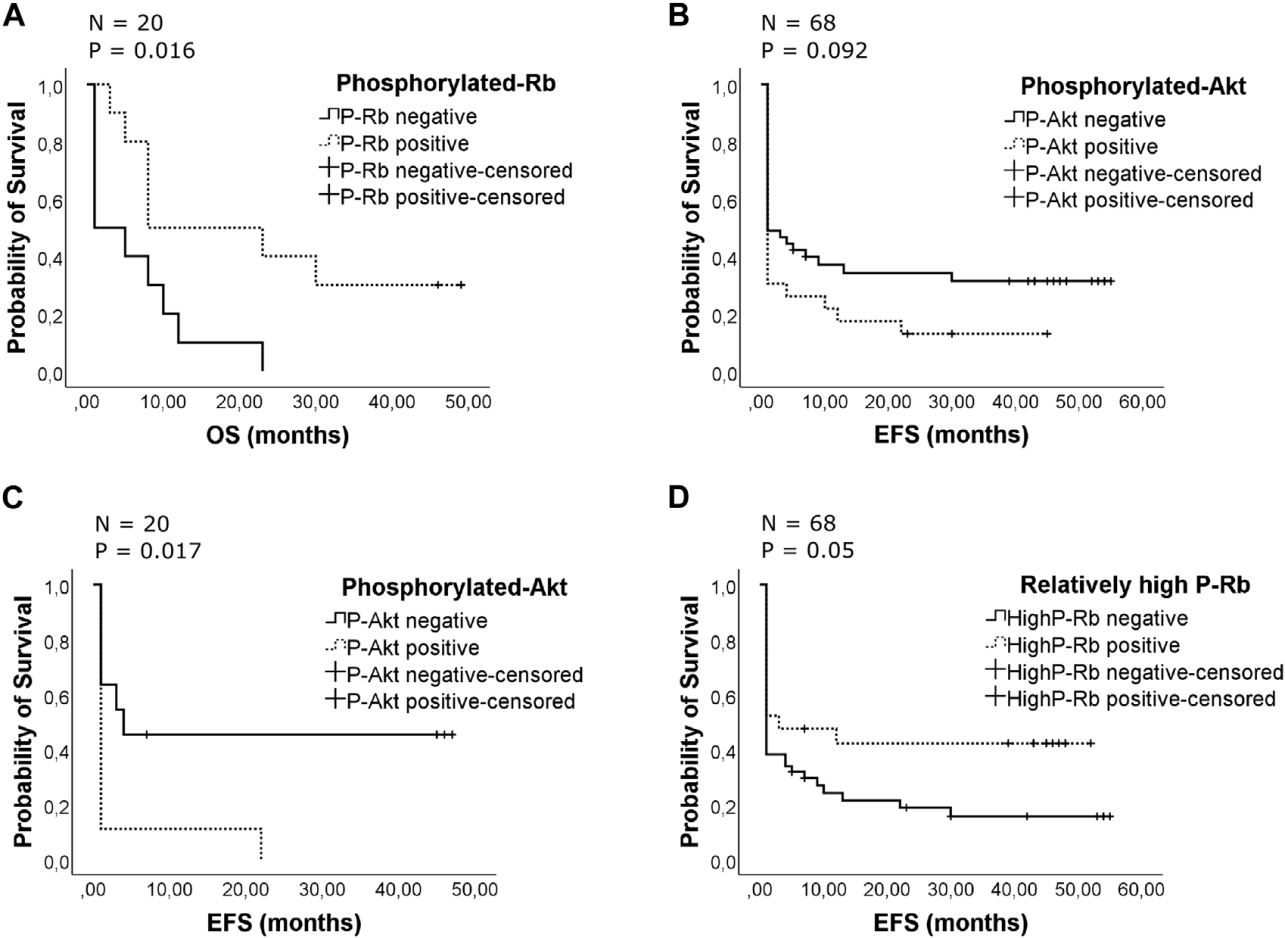
**(A)** Effect of the presence of phosphorylated retinoblastoma protein on patients’ overall survival with unfavorable cytogenetics. The Kaplan-Meier curves compare phosphorylated and unphosphorylated Rb groups for overall survival. The patients were censored at the time of death. Overall, there were 17 deaths, 7 from 10 P-Rb patients and 10 from 10 unphosphorylated patients. The median survival was 1 month in patients’ group with unphosphorylated Rb protein, while the median survival was 8 months in patients’ group with phosphorylated Rb (*p* = 0.016). **(B)** Effect of the presence of phosphorylated Akt on duration of event-free survival. The Kaplan-Meier curves compare phosphorylated and unphosphorylated Akt groups for event-free survival. The patients were censored at time of relapse or death or finding of failure to achieve CR. The survival for the whole studied patient population is shown. Overall, there were 50 events occurring in 20 from 23 P-Akt patients and 30 from 45 unphosphorylated ones. The mean survival was 8 months in patients’ group with phosphorylated Akt protein, while the mean survival was 19 months in patients’ group with unphosphorylated Akt (*p* = 0.092). **(C)** Effect of the presence of phosphorylated Akt on duration of event-free survival with unfavorable cytogenetics. The Kaplan-Meier curves compare phosphorylated and unphosphorylated Akt groups for event-free survival. The patients were censored at time of relapse or death or finding of failure to achieve CR. Overall, there were 15 events occurring in 9 from 9 P-Akt patients and 6 from 11 unphosphorylated patients. The mean survival was 3 months in patients’ group with phosphorylated Akt protein, while the mean survival was 22 months in patients’ group with unphosphorylated Akt (*p* = 0.017). **(D)** Effect of the presence of relatively high phosphorylated retinoblastoma protein on patients’ event-free survival. The Kaplan-Meier curves compare relatively high phosphorylated Rb groups with the others for event-free survival. The patients were censored at the time of relapse or death or finding of failure to achieve CR. Overall, there were 50 events, 12 from 21 relatively high P-Rb expression level patients and 38 from 47 patients with relatively low P-Rb or unphosphorylated Rb. The mean event-free survival was 23 months in patients’ group with relatively high phosphorylated Rb protein, while the mean event-free survival was 12 months in the other patients’ group (*p* = 0.05).

The patients studied had a median overall survival (OS) of 8 months with complete remission (CR) rates of 38% after first or second induction therapy (29% after first induction). The mean duration of event-free survival was 16 months. No statistically significant differences were found between the phosphorylated protein groups compared with their nonphosphorylated ones regarding median survival for all of the examined patients. No major differences in the cause of death were noted between the distinct groups. Surprisingly, we found phosphorylated Rb improve OS among patients with unfavorable cytogenetics (median 1 vs. 8 months, *p* = 0.016) (Figure 3A). Among patients with unfavorable cytogenetics phosphorylated-Rb represent a significant predictor of outcome examined by univariate (B = - 1.116; Exp(B) = 0.328; 95% CI for Exp(B) = 0.116–0.929; *p* = 0.036) and multivariate (B = - 1.465; Exp(B) = 0.231; 95% CI for Exp(B) = 0.06–0.885; *p* = 0.032) Cox proportional hazard regression analysis (data are not shown). Although the patients received a variety of therapeutic regimens, the type of chemotherapy was not a predictor of outcome among patients with unfavorable cytogenetics. Only the allogenic bone marrow transplantation (ABMT) represents a significant predictor of outcome (B = - 1.427; Exp(B) = 0.24; 95% CI for Exp(B) = 0.076–0.763; *p* = 0.016, data are not shown). Phospho-Rb remained a statistically significant independent predictor when ABMT category (yes or no) was added to the model (B = −1.645; Exp(B) = 0.193; 95% CI for Exp(B) = 0.053–0.707; *p* = 0.013). The summaries of multivariate regression analysis are shown in Supplementary Table S2.

The differences between groups in duration of event-free survival and the percentage of patients who achieved a complete remission were not statistically significant, although the phospho-Akt group showed inferior event-free survival compared to the others (mean 8 versus 19 months) but the difference was not significant (Figure 3B). Among patients with unfavorable cytogenetics presence of phosphorylated Akt represent significant worse EFS (mean 3 versus 22 months, *p* = 0.017) (Figure 3C). When CR status was analyzed, only 21% of the P-Akt positive patients achieved CR after the first induction, while it was only 11% among unfavorable cytogenetics patients (P = NS). The presence of phosphorylated Rb has a positive impact on event-free survival in unfavorable patients’ group (mean 5.5 vs. 16.6 months, results are not shown), but the differences were not statistically significant. When relative expression level was analyzed, relatively high phospho-Rb level provided a significant better EFS in full cohort of AML patients (mean 12 vs. 23 months, *p* = 0.05) (Figure 3D).

The presence of either phosphatase does not cause statistically significant difference in OS or in EFS, but we found the highest expression level of PHLPP provided significantly worse OS (median 1 vs. 10 months, *p* = 0.033) among full cohort of AML patients. If only PHLPP positive patients were analyzed, the highest PHLPP expression level worsenedoverall survival even more compared to others (median 1 vs. 23 months, 0.021). When overall survival time was analyzed among patients with Cox proportional hazard regression model, groups with both PTEN and PHLPP phosphatases represented significant predictors of outcome (B = −2.502; Exp(B) = 0.082; 95% CI for Exp(B) = 0.007–0.939; *p* = 0.044, data are not shown). It remained statistically significant predictor when ABMT category (yes or no) was added to the model (B = −2.939; Exp(B) = 0.053; 95% CI for Exp(B) = 0.004–0.636; *p* = 0.021). The summaries of the analysis are shown in Supplementary Table S3. When remission duration was analyzed only by age over 60 years at diagnosis it was an independent predictor (B = 1.174; Exp(B) = 3.234; 95% CI for Exp(B) = 1.371–7.628; *p* = 0.007, data are not shown).

The phosphoproteins detected at diagnosis usually disappear when the patient achieves complete remission with negative minimal residual disease. As an example, Western blot analysis of mononuclear cells separated from an AML patient the phosphorylated Rb detected at the time of diagnosis disappeared after induction treatment. The patient achieved minimal residual disease negative complete remission and proved to be a long-term survivor. The patient is still alive 10 years and 5 months after diagnosis (Figure 2 lane 5 and 6).

In some cases, phosphorylated proteins were not detectable at diagnosis but appeared after progression of disease. For example, Western blot analysis of mononuclear cells separated from an AML patient showed no phosphorylated protein at the time of diagnosis, but phosphorylated Rb and Akt protein appeared after progression of leukemia. There were 28% blast cells in the bone marrow at the diagnosis, and the patient achieved partial remission after induction treatment. There were 10% blast cells in the bone marrow by progression. The patient died in 3 months after the diagnosis due to septicemia and pneumonia (Figure 2 lane 7 and 8).

There was one MDS patient and one patient with paroxysmal nocturnal hemoglobinuria (PNH) whose first sample was taken before the appearance of AML and phosphorylated proteins were not detectable at that time. The second sample was taken when the MDS or PNH progressed to AML and phosphorylated Akt and Erk proteins were appeared in MDS patient (Figure 2 lane 9 and 10) and phosphorylated Akt were developed by PNH patient (Figure 2 lane 11 and 12) at the time of AML transformation. It suggests the pathogenic role of phosphoproteins in the development of AML.

### Phosphoprotein levels in MDS patients

Nine MDS patients (25%) had detectable phosphorylated Rb protein level. Akt and Erk protein phosphorylation was observed in five patients (15%). Nine from the 36 patients progressed to acute leukemia, and six from these patients showed at least one phosphorylated protein. For example, Western blot analysis of mononuclear cells separated from an MDS patient detected phosphorylated Rb and Akt protein. There were 1.6% blast cells in the bone marrow at the time of diagnosis. The patient progresses to AML in 11 months after diagnosis (Supplementary Figure S1.). There was no correlation between the Revised International Prognostic Scoring System (R-IPSS) score [58] and protein phosphorylation. Median survival was similar in the phosphorylated and unphosphorylated groups of patients. The median expression level of p-RB was significantly lower in MDS patients compared to AML patients (Independent Samples *t*-test, *p* = 0.045). Regarding the other proteins, there was no difference in the median expression levels between the MDS and AML patient groups.

## Discussion

Our present study shows that hyperphosphorylation of Rb, Akt or Erk proteins is a common event in the development of acute myeloid leukemia. Other research groups have also analyzed the occurrence of phosphoproteins in myeloid blast cells and concluded that hyperphosphorylation of signaling proteins affect negatively the clinical outcome of AML [46, 71, 72]. We have demonstrated that 69% of AML patients show at least one constitutive phosphorylated form of the above (Rb, Akt and Erk) examined proteins. The appearance of phosphoproteins simultaneously with the development or progression of AML confirm the key role of protein phosphorylation in the pathogenesis of leukemia and the progression from MDS to AML. The clonal evolution may explain the development of phosphorylation of proteins in leukemic blast cells during the course of disease. This process can select one or more phosphorylated proteins in blasts, which seems to provide survival advantage against cells negative for phosphorylated proteins. The existence of two or three phosphorylated protein at the same time represent worse event-free survival. The presence of phosphorylated Akt protein alone reduces the EFS among AML patients. The difference is significant in patients with unfavorable cytogenetics. Overall, the vast majority of patients with phosphorylated Akt relapsed or died (91.3%, P = NS). Phospho-Akt protein was detectable in 34.7% of AML patients. Min et al. demonstrated constitutive phosphorylation of Akt on Ser473 residue in more than 70% of blast cells separated from AML patients with significantly shorter overall survival compared to patients without phosphorylated Akt. Similar to our findings, there were no significant differences in Akt phosphorylation regarding FAB subtype, cytogenetic abnormalities or white blood cell counts [71].

PTEN and PHLPP phosphatases, the negative regulators of the PIP3K/Akt pathway, have also been investigated. Previous reports indicated significantly better outcome in PHLPP expressing solid tumors [73, 74]. In contrast, Ono et al. found that high PHLPP expression in AML blast cells is associated with poor prognosis [75]. Several investigators presented lower PTEN mRNA and protein level in AML patients compared with healthy people, and it correlates positively with poor prognostic factors [76, 77]. Huang et al. demonstrated that PTEN positive elderly patients with refractory AML tended to have an improved prognosis compared to PTEN negative patients [78]. In our study, the presence of either phosphatase did not show a significant impact on survival in our AML patients.

We found a significant correlation between the FAB5 subtype and the presence of both PHLPP and PTEN and PHLPP alone. Although the number of patients in the FAB5 group is small, these data suggest a possible link between the PHLPP and PTEN pathway and myeloid development or monocytic differentiation, respectively. This is supported by the well-known role of the PI3K/Akt pathway in monocyte differentiation [79, 80]. FAB5 maturation state in AML is of particular interest because in a recent publication Pei S et al demonstrated that the monocyte morphology was found highly significant to be associated with a disease that was refractory to the combination treatment of hypomethylating agent Azacitidine and the highly specific Bcl-2 inhibitor Venetoclax [81]. The investigators proved that monocytic leukemia blasts lose expression of the Venetoclax target Bcl-2 while Myeloid cell leukemia‐1 (MCL-1) protein, an antiapoptotic member of the Bcl-2 family [82] expression is significantly higher in FAB-M5 patients. The exact role of the PTEN and PHLPP in the emergence of Venetoclax resistance requires further investigation. Nevertheless, the role of the Akt signaling pathway possibly by upregulation of non-Bcl-2 antiapoptotic proteins such as MCL-1 and Bcl-XL in the development of venetoclax resistance has been confirmed in hematologic malignancies [83–85]. In addition, treatment with inhibitors acting on the Akt signaling pathway helps restore Venetoclax sensitivity in leukemic cells [86]. Clarifying the role of PTEN and PHLPP protein in Venetoclax resistance and monocyte differentiation may be of great importance even in terms of therapeutic decisions. This subgroup of AML patients benefits from MLC-1 inhibitors in addition to chemotherapy treatment compared to Venetoclax [81,83].

We observed Erk phosphorylation in 26% of samples from our patients. Authors from MD Anderson Cancer Centre demonstrated elevated phospho-Erk level via a flow cytometric technique in more than 80% of AML patients. The authors found no correlation between constitutive Erk phosphorylation and the course of disease [72]. The higher occurrence rate of phospho-Erk protein demonstrated by these authors compared with our result (26%) might be explained with better sensitivity of flow cytometric technique compared with Western blots.

Contradictory data exist about the impact of retinoblastoma protein on survival. For example, Kornblau et al. found phosphorylated Rb protein in 15% of blast cells from AML patients with significant shorter survival compared with patients without hyperphosphorylated Rb [46]. The difference between this previous study and our data (15% versus 46.3%) may be due to the fact that our group studied Rb protein phosphorylated on Thr821/826 sites, while Kornblau et al. investigated maximally phosphorylated Rb. Also, Kornblau et al. found low retinoblastoma protein level associated with a significantly worse outcome among AML patients [3]. In contrast, more and more data suggest that loss of Rb function increases chemosensitivity in different type of cancer. For example, Zagorski et al. presented this finding in lung cancer cell lines [87]. Derenzini et al. demonstrated superior disease-free survival in human breast cancer cells with lack of retinoblastoma protein expression [88]. In accordance with those findings, Treré et al. found better outcome among triple-negative breast cancer patients with retinoblastoma protein negative compared to retinoblastoma positive patients [89]. Presumably, loss of retinoblastoma protein or functionally inactivated phosphorylated form may provide greater chemosensitivity and consequently better outcome among treated patients. Greater chemosensitivity may explain better OS among our AML patients with unfavorable cytogenetics, but establishing an exact mechanism requires further investigation. These conflicting data highlight the complexity concerning the regulation of the process of leukemogenesis by protein phosphorylation and the impacts on survival. Another explanation for better outcome with P-Rb is the lower leukocyte count at the time of diagnosis in patients with phosphorylated Rb. 90% of the P-Rb positive patients had less than 30 G/L leukocyte count by diagnosis in patients’ group with unfavorable cytogenetics (P = NS).

The phosphorylated form of the investigated proteins is present in mononuclear cells separated from MDS patients. Our results suggest that constitutive phosphorylation can be responsible for progression from MDS to AML. In accordance with our finding a recent publication by Zheng Z et al. [90] demonstrated patients with MDS or MDS-AML presented elevated levels of phosphorylated Erk and phosphorylated Akt proteins. The authors confirmed the role of the MEK/Erk and PI3K/Akt signaling pathway in the transformation of MDS to AML by modulating histone methylation via the trimethylation of H3 on lysine 27 (H3K27me3) methylase and demethylase pathways and by regulating distal-less homeobox 5 (DLX5) gene transcription which gene has previously determined to have an anti-tumor effect in AML an MDS [91, 92]. Another investigator examined phosphoproteins in MDS patients too. For example, Nyakern et al. observed high levels of phospho-Ser473-Akt in 90% of mononuclear cells separated from patients diagnosed with high-risk MDS. In contrast, low-risk MDS patients exhibited low level or absence of phosphorylated Akt protein [93]. In our study, we did not find correlation between the MDS risk score and presence of phosphorylated proteins.

Most patients with acute myeloid leukemia, especially patients with complex cytogenetics develop refractory or relapsed disease even in the presence of novel therapeutic agents and combined chemotherapy. For this reason, it is particularly important to develop new agents that target different mechanisms of leukemogenesis - specific genetic abnormalities or mutant proteins - and their personalized application can improve long-term remission. Numerous kinase inhibitors against constitutively activated cell signaling pathways have been developed. The agents listed below targeting the Akt or Erk signaling pathways are currently in various phases of active clinical trials or have been available in recently completed studies. The Raf inhibitor Sorafenib [94], the MEK inhibitor GDC-0973 (Cobimetinib) [95], the ERK1/2 inhibitor LY3214996 (Temuterkib) [96], a pan-class I PI3K inhibitor Rigosertib [97], Akt inhibitor MK2206 [98] and Akt/Erk inhibitor ONC201 [99] may be accessible for AML patients within the framework of clinical trials alone or in combination with other chemotherapeutic agents. Several clinical trials have been reported that the combination of hypomethylating agents (HMAs) Azacitidine or Decitabine and Venetoclax can increase the response rates and the overall survival for older or unfit newly diagnosed AML patients who are not suitable for intensive chemotherapy [100, 101]. These findings resulted in the United States Food and Drug Administration’s (FDA) approval of this combination as a standard of care for this population. In addition, azacitidine has a place in the maintenance treatment [102] and in the treatment of relapsed or refractory AML in combination with Venetoclax or as a supplement to salvage chemotherapy [103, 104]. According to a recently published recommendation, venetoclax-based regimen is preferable in young adult leukemia patients too [105]. Patients with adverse genetic abnormalities (especially abnormalities of chromosome 5, 7, or 17 [106, 107], and mutated TP53 [108]) may benefit mostly from HMA treatment, and supplementing the therapy with Venetoclax is particularly recommended [109], although the treatment of patients with TP53 mutation remains a major challenge. The addition of Venetoclax to the therapeutic regimens may be of particular interest in p-Akt positive patients because it is known that Akt upregulates Bcl-2 expression [30] Beside the Venetoclax [110], BP1002, a liposomal Bcl-2 antisense oligodeoxynucleotide [111] is still available now in a clinical trial for AML patients. Furthermore, Akt activates Mdm2 [27], therefore Mdm2 antagonists, for example, Milademetan [100] and AMG-232 (Navtemadlin) [112] in the case of AML patients expressing phosphorylated Akt may be especially reasonable choices.

The role of DNA methylation [113, 114] and activation of the Akt and Erk signaling pathways [115, 116] in the development of chemoresistance is widely investigated. There is considerable evidence that both processes contribute to reduced sensitivity to chemotherapy agents. Thus, by attacking the leukemic cells at two points, therapeutic response can be improved in p-Akt and p-Erk positive patients with a combination of HMAs and kinase inhibitors acting on the Akt and Erk signaling pathway.

Limitations of our study are the low number of cases, in some patients missing FLT3, nucleophosmin, and cytogenetics data, and the limited amount of samples. Resulting from this, each experiment could be repeated only a few times.

In summary, we identified subgroups of AML patients with hyperphosphorylated proteins with Western blot technique. Akt phosphorylation seems to have a negative impact on event-free survival, especially in patients with unfavorable cytogenetics. The presence of phosphorylated retinoblastoma improves the overall survival in the patients’ group with unfavorable cytogenetics. The exact molecular mechanisms how these phosphorylated proteins influence survival is not clear, but presumably, patients with phosphorylated proteins may profit from targeted treatments especially kinase inhibitors supplemented with the standard therapy. Although our results need to be confirmed on a larger number of patients, it appears that in P-Akt positive patients more emphasis should be placed on targeted therapy and chemotherapy supplemented with HMA or Venetoclax respectively and they may be candidates for an early bone marrow transplantation because of unfavorable outcome and frequent relapse rate. The preference for the method applied here is that in the case of measurable peripheral leukemic blasts, it can be useful to specify the phosphorylated proteins without unpleasant bone marrow aspiration. In selected cases, it could be suitable for monitoring remission, relapse, or progression of disease.

## Figures and Tables

**Table:** 

**ABMT**	Allogenic bone marrow transplantation
**AML**	Acute myeloid leukemia
**APL**	Acute promyelocytic leukemia
**Ara-C**	Cytosine arabinoside
**Bad**	Bcl-2 Associated Agonist Of Cell Death
**Bax**	Bcl-2-associated X protein
**BCA**	Bicinchoninic acid
**Bcl-2**	B-cell lymphoma 2
**cAMP**	Cyclic Adenosine Monophosphate
**CDK**	Cyclin dependent kinase
**CR**	Complete remission
**CREB**	cAMP response element-binding protein
**ECL**	Enhanced chemiluminescence
**EFS**	Event free survival
**Erk**	extracellular signal-regulated kinases
**FAB**	French-American-British classification
**FLT-3**	Fms-like tyrosine kinase 3
**FOXO**	Forkhead box O
**GAPDH**	Glyceraldehyde-3-phosphate-dehydrogenase
**GSK-3β**	glycogen synthase kinase-3beta
**HDAC**	Histone deacetylase
**HMAs**	Hypomethylating agents
**ITD**	Internal tandem duplication
**MAPKs**	Mitogen-activated protein kinases
**MCL-1**	Myeloid cell leukemia‐1
**Mdm2**	Mouse double minute 2
**MDS**	Myelodysplastic syndrome
**MKPs**	Mitogen-activated protein kinase phosphatases
**MPAL**	Mixed phenotype acute leukemia
**MRC**	Myelodysplasia-related changes
**mTOR**	Mammalian target of rapamycin
**NFκB**	Nuclear factor kappa-light-chain-enhancer of activated B cells
**NMPc**	Cytoplasmic nucleophosmin
**OS**	Overall survival
**P-Akt**	Phosphorylated-Akt
**PBS**	Phosphate-buffered saline
**PDK1/2**	Phosphoinositide-dependent protein kinase 1/2
**P-Erk**	Phosphorylated- extracellular signal-regulated kinases
**PHLPP**	PH domain and leucine-rich repeat Protein Phosphatase
**PIP3**	Phosphatidylinositol (3,4,5)-triphosphate
**PIP3K**	Phosphoinositide 3-kinases
**PKB**	Protein kinase B
**PNH**	Paroxysmal nocturnal hemoglobinuria
**PP1**	Protein phosphatase-1
**PP2A**	Protein phosphatase-2A
**P-Rb**	Phosphorylated-retinoblastoma
**PTEN**	Phosphatase and tensin homolog
**Rb**	Retinoblastoma
**R-IPSS**	Revised International Prognostic Scoring System
**SDS**	Na-dodecyl sulfate
**TBS**	Tris-Buffered Saline
**TKD**	Tyrosine kinase domain mutations
**WBC**	White blood cell
**WHO**	World Health Organization

## Data Availability

The original contributions presented in the study are included in the article/Supplementary Material, further inquiries can be directed to the corresponding author.
