# Supplementary material for: The roles of phosphorylation of signaling proteins in the prognosis of acute myeloid leukemia
Source: Pathol Oncol Res. 2024 Jul 5;30:1611747. doi: 10.3389/pore.2024.1611747 (PMC11257863; doi:10.3389/pore.2024.1611747)
Supplement: Supplementary file 1 [file DataSheet1.docx]

**Supplementary Material**


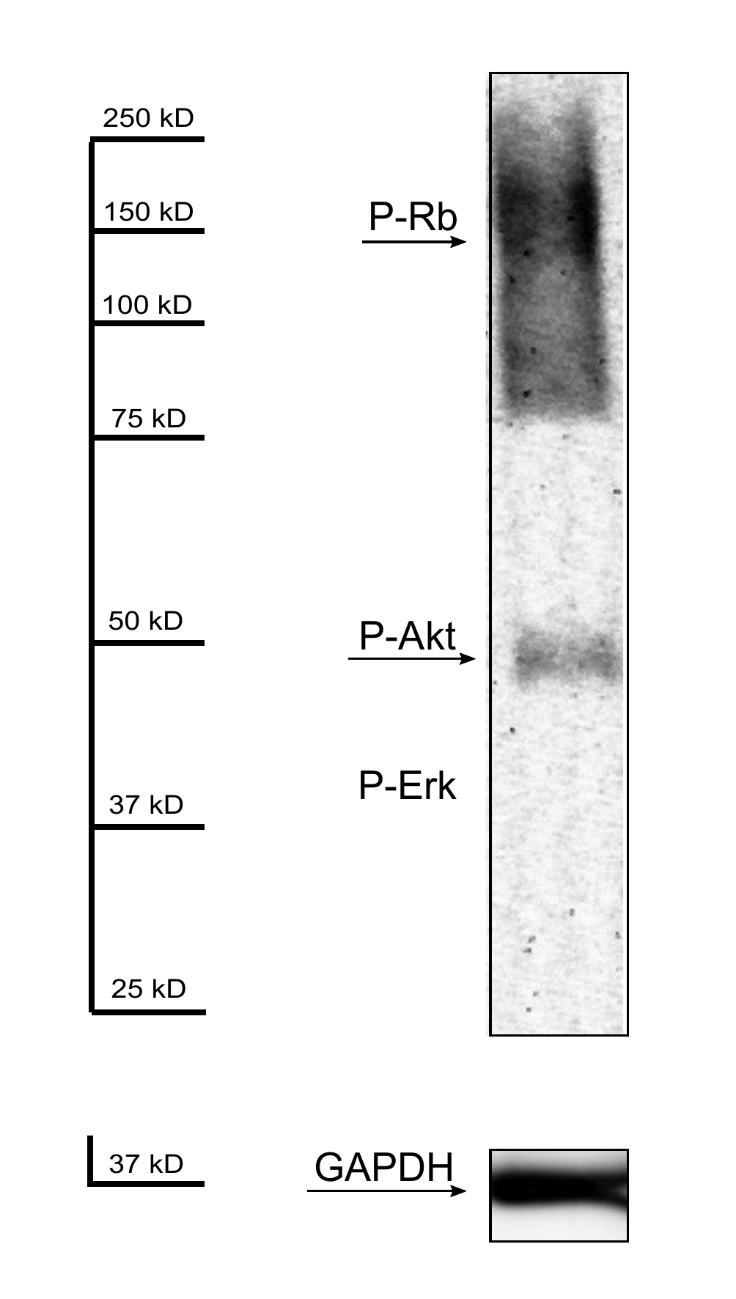


**Supplementary Figure 1.** Western blots of P-Rb, P-Akt and P-Erk of mononuclear cells separated from bone marrow of an MDS patient.

**Suppl. Table 1.** Applied treatment regimens of AML patients

| **Induction treatment** | "3+7" | 43 |
| --- | --- | --- |
|  | AIDA protocol | 2 |
|  | Flag-Ida | 2 |
| **Consolidation treatment** | High-dose Ara-C | 27 |
| **Salvage treatment** | Flag-Ida | 7 |
|  | Flag | 17 |
|  | HAM | 11 |
|  | MEC | 2 |
|  | FLAMSA | 1 |
|  | CLAG-M | 1 |
|  | Clofarabine | 5 |
| **Palliative** | Reduce dose "3+7" | 15 |
|  | Ara-C alone | 8 |
|  | Hydroxyurea | 17 |
|  | Only Supportive | 4 |
| Allogenic transplantation | | 16 |

**Suppl. Table 2.** Summary of multivariate Cox proportional hazards regression model for patients with unfavorable cytogenetics.*

|  | P | HR | 95.0% CI for HR | |
| --- | --- | --- | --- | --- |
|  |  |  | Lower | Upper |
| Phosphorylated Rb | 0.013 | 0.193 | 0.053 | 0.707 |
| ECOG01 | 0.742 | 0.751 | 0.136 | 4.146 |
| ECOG45 | 0.327 | 2.606 | 0.383 | 17.717 |
| AgeOver60years | 0.571 | 0.583 | 0.090 | 3.767 |
| AMLwithMRC | 0.609 | 1.349 | 0.428 | 4.254 |
| ABMT | 0.015 | 0.148 | 0.031 | 0.695 |

*Overall survival time defined as the endpoint and the model included terms for Rb category: phosphorylated Rb present (risk group) or absent (baseline group), ECOG performance status: 0-1 present (risk group) or absent (baseline group) and 4-5 present (risk group) or absent (baseline group), age: more than 60 years old (risk group) or not (baseline group), WHO classification: AML with MRC (risk group) or not (baseline group), ABMT: yes (risk group) or no (baseline group). Phosphorylated-Rb was a significant independent predictor of outcome.

**Suppl.Table 3.** Summary of Cox multivariate proportional hazards regression model among AML patients.*

|  | P | HR | 95.0% CI for HR | |
| --- | --- | --- | --- | --- |
|  |  |  | Lower | Upper |
| PTEN | 0.105 | 2.128 | 0.853 | 5.309 |
| PHLPP | 0.104 | 6.919 | 0.671 | 71.296 |
| BothPhosphatases | 0.021 | 0.053 | 0.004 | 0.636 |
| ECOG01 | 0.965 | 1.022 | 0.392 | 2.662 |
| ECOG45 | 0.151 | 3.572 | 0.629 | 20.297 |
| AgeOver60years | 0.320 | 1.635 | 0.621 | 4.308 |
| AMLwithMRC | 0.324 | 0.623 | 0.243 | 1.597 |
| Unfavorable | 0.075 | 2.132 | 0.927 | 4.902 |
| ABMT | 0.003 | 0.198 | 0.068 | 0.577 |

*Overall survival time defined as the endpoint and the model included terms for PTEN category: present (risk group) or absent (baseline group), PHLPP category: present (risk group) or absent (baseline group), both phosphatases category: present (risk group) or absent (baseline group), ECOG performance status: 0-1 present (risk group) or absent (baseline group) and 4-5 present (risk group) or absent (baseline group), age: more than 60 years old (risk group) or not (baseline group), WHO classification: AML with MRC (risk group) or not (baseline group), cytogenetics: unfavorable (risk group) or not (baseline group), ABMT: yes (risk group) or no (baseline group). Presence of both phosphatases represent a significant predictor of outcome.

**Figure 1, first panel.** The membrane was cut into 3 parts above the standard band indicating 75 kDa molecular weight and below the band indicating 50 kDa molecular weight. The arrows indicate where the membrane was cut. The upper part of the membrane was incubated with anti-human Retinoblastoma primary antibody, the middle part of the membrane was incubated with anti-human Akt primary antibody, the lower part of the membrane was incubated with anti-human Erk primary antibody. The lower part of the membrane was stripped and the membrane was incubated with anti-human GAPDH antibody (GAPDH was not shown in the figure).





Incubated with anti-Erk antibody

Incubated with anti-Akt antibody

Incubated with anti-Rb antibody





Incubated with anti-GAPDH antibody

**Figure 1, first panel.** The membrane was cut into 3 parts above the standard band indicating 75 kDa molecular weight and below the band indicating 50 kDa molecular weight. The arrows indicate where the membrane was cut. The upper part of the membrane was incubated with anti-human PHLPP primary antibody, the middle part of the membrane was incubated with anti-human PTEN primary antibody, the lower part of the membrane was incubated with anti-human GAPDH primary antibody.





Incubated with anti-PHLPP antibody

Incubated with anti-PTEN antibody





Incubated with anti-GAPDH antibody

**Figure 1, second panel.** The membrane was cut into 3 parts above the standard band indicating 75 kDa molecular weight and below the band indicating 50 kDa molecular weight. The arrows indicate where the membrane was cut. The upper part of the membrane was incubated with anti-human phospho-Thr821/826-Rb primary antibody, the middle part of the membrane was incubated with anti-human phospho-Ser473-Akt primary antibody, the lower part of the membrane was incubated with anti-human phospho-Thr202/Tyr404-Erk primary antibody. The lower part of the membrane was stripped and the membrane was incubated with anti-human GAPDH antibody.





Incubated with anti-P-Erk antibody

Incubated with anti-P-Akt antibody

Incubated with anti-P-Rb antibody





Incubated with anti-GAPDH antibody

**Figure 2, lane 1 and 2.** The membrane was cut into 3 parts above the standard band indicating 75 kDa molecular weight and below the band indicating 50 kDa molecular weight. The arrows indicate where the membrane was cut. The upper part of the membrane was incubated with anti-human phospho-Thr821/826-Rb primary antibody, the middle part of the membrane was incubated with anti-human phospho-Ser473-Akt primary antibody, the lower part of the membrane was incubated with anti-human phospho-Thr202/Tyr404-Erk primary antibody. The lower part of the membrane was stripped and the membrane was incubated with anti-human GAPDH antibody.





Incubated with anti-P-Erk antibody

Incubated with anti-P-Akt antibody

Incubated with anti-P-Rb antibody





Incubated with anti-GAPDH antibody

**Figure 2, lane 3 and 4.** The membrane was cut into 3 parts above the standard band indicating 75 kDa molecular weight and below the band indicating 50 kDa molecular weight. The arrows indicate where the membrane was cut. The upper part of the membrane was incubated with anti-human phospho-Thr821/826-Rb primary antibody, the middle part of the membrane was incubated with anti-human phospho-Ser473-Akt primary antibody, the lower part of the membrane was incubated with anti-human phospho-Thr202/Tyr404-Erk primary antibody. The lower part of the membrane was stripped and the membrane was incubated with anti-human GAPDH antibody.

Incubated with anti-P-Rb antibody





Incubated with anti-GAPDH antibody

Incubated with anti-P-Erk antibody

Incubated with anti-P-Akt antibody





**Figure 2, lane 5.** The membrane was cut into 3 parts above the standard band indicating 75 kDa molecular weight and below the band indicating 50 kDa molecular weight. The arrows indicate where the membrane was cut. The upper part of the membrane was incubated with anti-human phospho-Thr821/826-Rb primary antibody, the middle part of the membrane was incubated with anti-human phospho-Ser473-Akt primary antibody, the lower part of the membrane was incubated with anti-human phospho-Thr202/Tyr404-Erk primary antibody. The lower part of the membrane was stripped and the membrane was incubated with anti-human GAPDH antibody.





Incubated with anti-P-Erk antibody

Incubated with anti-P-Akt antibody

Incubated with anti-P-Rb antibody





Incubated with anti-GAPDH antibody

**Figure 2, lane 6.** The membrane was cut into 3 parts above the standard band indicating 75 kDa molecular weight and below the band indicating 50 kDa molecular weight. The arrows indicate where the membrane was cut. The upper part of the membrane was incubated with anti-human phospho-Thr821/826-Rb primary antibody, the middle part of the membrane was incubated with anti-human phospho-Ser473-Akt primary antibody, the lower part of the membrane was incubated with anti-human phospho-Thr202/Tyr404-Erk primary antibody. The lower part of the membrane was stripped and the membrane was incubated with anti-human GAPDH antibody.





Incubated with anti-P-Erk antibody

Incubated with anti-P-Akt antibody

Incubated with anti-P-Rb antibody





Incubated with anti-GAPDH antibody

**Figure 2, lane 7 and 8.** The membrane was cut into 3 parts above the standard band indicating 75 kDa molecular weight and below the band indicating 50 kDa molecular weight. The arrows indicate where the membrane was cut. The upper part of the membrane was incubated with anti-human phospho-Thr821/826-Rb primary antibody, the middle part of the membrane was incubated with anti-human phospho-Ser473-Akt primary antibody, the lower part of the membrane was incubated with anti-human phospho-Thr202/Tyr404-Erk primary antibody. The lower part of the membrane was stripped and the membrane was incubated with anti-human GAPDH antibody.





Incubated with anti-P-Erk antibody

Incubated with anti-P-Akt antibody

Incubated with anti-P-Rb antibody





Incubated with anti-GAPDH antibody

**Figure 2, lane 9.** The membrane was cut into 3 parts above the standard band indicating 75 kDa molecular weight and below the band indicating 50 kDa molecular weight. The arrows indicate where the membrane was cut. The upper part of the membrane was incubated with anti-human phospho-Thr821/826-Rb primary antibody, the middle part of the membrane was incubated with anti-human phospho-Ser473-Akt primary antibody, the lower part of the membrane was incubated with anti-human phospho-Thr202/Tyr404-Erk primary antibody. The lower part of the membrane was stripped and the membrane was incubated with anti-human GAPDH antibody.





Incubated with anti-P-Erk antibody

Incubated with anti-P-Akt antibody

Incubated with anti-P-Rb antibody





Incubated with anti-GAPDH antibody

**Figure 2, lane 10.** The membrane was cut into 3 parts above the standard band indicating 75 kDa molecular weight and below the band indicating 50 kDa molecular weight. The arrows indicate where the membrane was cut. The upper part of the membrane was incubated with anti-human phospho-Thr821/826-Rb primary antibody, the middle part of the membrane was incubated with anti-human phospho-Ser473-Akt primary antibody, the lower part of the membrane was incubated with anti-human phospho-Thr202/Tyr404-Erk primary antibody. The lower part of the membrane was stripped and the membrane was incubated with anti-human GAPDH antibody.





Incubated with anti-P-Erk antibody

Incubated with anti-P-Akt antibody

Incubated with anti-P-Rb antibody





Incubated with anti-GAPDH antibody

**Figure 2, lane 11.** The membrane was cut into 3 parts above the standard band indicating 75 kDa molecular weight and below the band indicating 50 kDa molecular weight. The arrows indicate where the membrane was cut. The upper part of the membrane was incubated with anti-human phospho-Thr821/826-Rb primary antibody, the middle part of the membrane was incubated with anti-human phospho-Ser473-Akt primary antibody, the lower part of the membrane was incubated with anti-human phospho-Thr202/Tyr404-Erk primary antibody. The lower part of the membrane was stripped and the membrane was incubated with anti-human GAPDH antibody.





Incubated with anti-P-Erk antibody

Incubated with anti-P-Akt antibody

Incubated with anti-P-Rb antibody





Incubated with anti-GAPDH antibody

**Figure 2, lane 12.** The membrane was cut into 3 parts above the standard band indicating 75 kDa molecular weight and below the band indicating 50 kDa molecular weight. The arrows indicate where the membrane was cut. The upper part of the membrane was incubated with anti-human phospho-Thr821/826-Rb primary antibody, the middle part of the membrane was incubated with anti-human phospho-Ser473-Akt primary antibody, the lower part of the membrane was incubated with anti-human phospho-Thr202/Tyr404-Erk primary antibody. The lower part of the membrane was stripped and the membrane was incubated with anti-human GAPDH antibody.





Incubated with anti-P-Erk antibody

Incubated with anti-P-Akt antibody

Incubated with anti-P-Rb antibody





Incubated with anti-GAPDH antibody

**Supplementary Figure 1.** The membrane was cut into 3 parts above the standard band indicating 75 kDa molecular weight and below the band indicating 50 kDa molecular weight. The arrows indicate where the membrane was cut. The upper part of the membrane was incubated with anti-human phospho-Thr821/826-Rb primary antibody, the middle part of the membrane was incubated with anti-human phospho-Ser473-Akt primary antibody, the lower part of the membrane was incubated with anti-human phospho-Thr202/Tyr404-Erk primary antibody. The lower part of the membrane was stripped and the membrane was incubated with anti-human GAPDH antibody.





Incubated with anti-P-Erk antibody

Incubated with anti-P-Akt antibody

Incubated with anti-P-Rb antibody





Incubated with anti-GAPDH antibody
